# Dynamic Language Network in Early and Late Cantonese–Mandarin Bilinguals

**DOI:** 10.3389/fpsyg.2020.01189

**Published:** 2020-06-18

**Authors:** Xiaojin Liu, Liu Tu, Xiaoxi Chen, Miao Zhong, Meiqi Niu, Ling Zhao, Zhi Lu, Ruiwang Huang

**Affiliations:** ^1^Center for the Study of Applied Psychology, Key Laboratory of Mental Health and Cognitive Science of Guangdong Province, School of Psychology, South China Normal University, Guangzhou, China; ^2^College of Foreign Studies, Jinan University, Guangzhou, China; ^3^School of Management, Jinan University, Guangzhou, China; ^4^Guangdong Collaborative Innovation Center for Language Research and Services, Guangdong University of Foreign Studies, Guangzhou, China

**Keywords:** age of acquisition, dynamic functional connectivity, second language, bilingual experience, bi-dialects

## Abstract

The brain representation of language in bilinguals is sculptured by several factors, such as age of acquisition (AoA) and proficiency level (PL) in second language. Although the effect of AoA-L2 on brain function and structure has been studied, little attention is devoted to dynamic properties of the language network and their differences between early and late bilinguals. In this study, we acquired resting-state fMRI data from early and late Cantonese (L1)–Mandarin (L2) bilinguals with high PLs of verbal fluency in both languages. We then analyzed dynamic functional connectivity (dFC) by using the sliding-windows approach, estimated the dFC states by using the *k*-means clustering algorithm, and calculated the dynamic topological properties of the language network for the early and late bilinguals. We detected four dFC states, State 1, State 2, State 3, and State 4, which may be related to phonetic processing, semantic processing, language control, and syntactic processing, respectively. Compared to the late bilinguals, the early bilinguals showed higher dFC between the inferior frontal area and the temporal area in State 1 and State 2, while higher dFC between the cerebellum and other regions in State 3. The early bilinguals showed a higher clustering coefficient and local and global efficiency in State 1 and State 3, but lower characteristic path length in State 1, than the late bilinguals. Together, these results suggested that AoA-L2 affects temporal neural activation and dynamic topological properties of the language network. These findings provide new information to understand the effect of experience of L2 acquisition on language network in bilinguals.

## Introduction

Bilingual brains can be shaped by age of acquisition in second language (AoA-L2) and proficiency level in L2 (PL-L2) (Abutalebi, 2008). Early bilinguals are more likely to use a common neural mechanism to process L1 and L2 (Klein et al., 2006; Garbin et al., 2011), while late bilinguals with low PL-L2 may recruit more activation of cognitive control regions when processing L2 (Saur et al., 2009; Berken et al., 2015). Language processing is supported by a set of brain regions located in the language network (Price, 2010; Tie et al., 2014; Seo et al., 2018), in which the information transfers as a dynamic instead of static signal (Friederici and Gierhan, 2013; Fedorenko and Thompson-Schill, 2014; Wu et al., 2019). The dynamic property of the language network is mainly reflected in regional activation of the language network changing over time when subject processing language or even in brain resting state (Fedorenko and Thompson-Schill, 2014; Chai et al., 2016). Although previous studies (Wartenburger et al., 2003; Hernandez et al., 2007; Berken et al., 2015) detected a brain language network in early and late bilinguals, a few studies reported the effect of AoA-L2 on the dynamic properties of the language network in bilinguals.

Previous studies (Klein et al., 2014; Wei et al., 2015; Hämäläinen et al., 2017; Mitsuhashi et al., 2020; Ou et al., 2020) examined the effect of AoA-L2 on brain intrinsic FC, structural morphology, and task-based activation in bilinguals. Gullifer et al. (2018) selected the anterior cingulate (BA 24), the left caudate, the inferior frontal gyrus (IFG, BA 44, and BA 47), and the inferior parietal lobule (IPL, BA 40) as seeds to study the impact of AoA-L2 on resting-state FC in French–English bilinguals. They found that earlier AoA-L2 related to higher FC between left and right IFG, and to reduced reliance on proactive executive control during the completion of an AX-Continuous Performance Task outside the MRI scanner. These results suggested that different experiences of L2 acquisition impact brain intrinsic FC patterns and neural network involved in the executive control. Mechelli et al. (2004) studied the difference in cortical thickness of brain regions between early and late bilinguals. They found that late bilinguals showed increased cortical thickness in the IFG and decreased cortical thickness in the right IFG compared to early bilinguals. The above studies indicated that the AoA-L2 influences brain functional and structural neuroplasticity in bilinguals.

However, the effect of AoA-L2 on the dynamic properties of the language network is still unclear. Actually, the language network is a dynamic system (Blumstein and Amso, 2013; Fedorenko and Thompson-Schill, 2014; Chai et al., 2016). On the one hand, several crucial regions predominantly located in the left hemisphere, such as the IFG, the MFG, and anterior temporal regions, consistently coactivate with each other. On the other hand, other regions mainly located in the right hemisphere, such as middle anterior and posterior temporal regions, coactivate with others at different times during language processing and at brain resting state. Moreover, previous studies (Hosoda et al., 2013; Yang et al., 2015) found that the changed FC within the language network was related to L2 learning, indicating the dynamic properties of the language network in some degree. Yang et al. (2015) recruited 39 native English speakers to learn a novel tonal vocabulary and examined neural activity associated with L2 word learning. After a period of 6 weeks in training session, they found higher FC among the IFG, the middle frontal gyrus (MFG), the supplementary motor area (SMA), the insula (INS), the superior temporal gyrus (STG), and the IPL in successful learners compared to less successful learners. These findings reflected that the dynamic properties of the language network could be detected during brain resting state, language processing, and language training. However, most previous studies only considered the entire rs-fMRI scanning the static but not dynamic properties to analyze the effect of AoA-L2 on brain functional neuroplasticity. Revealing the dynamic properties of the language network may provide us a novel perspective to understand the neural mechanisms in different experience of L2 acquisition.

In this study, we aimed to investigate the effect of AoA-L2 on the dynamic properties of the language network in Cantonese–Mandarin bilinguals with high PL-L2. We analyzed dynamic FC (dFC) by using the sliding-windows approach and estimated the dynamic topological properties of the language network by using the graph theory. The graph theory has been widely used to measure the topological properties of brain networks (Wang et al., 2010; Bullmore and Bassett, 2011; Karwowski et al., 2019). For example, Sulpizio et al. (2020) applied the graph theory to explore the neural activation within the language and control networks in bilinguals. In this study, we selected four parameters, clustering coefficient (*C*_w_), characteristic path length (*L*_w_), global efficiency (*E*_glob_), and local efficiency (*E*_loc_), to characterize the dynamic topological properties of the language network. These four parameters can be used to examine the local (*C*_w_, *E*_loc_) and global (*L*_w_, *E*_glob_) information communication in the network and provide the altered information transferring within the language network. Since Broca’s area and Wernicke’s area are considered as core regions in language processing (Kim et al., 1997; Tomasi and Volkow, 2012; Zhu et al., 2014), we constructed the language network in early and late bilinguals separately by selecting the seeds in Broca’s area and Wernicke’s area. The dFC was calculated with the sliding-windows approach (Chang and Glover, 2010). Previous studies (Consonni et al., 2013; Sabourin et al., 2014; Archila-Suerte et al., 2015) showed that different neural activation during language processing could be detected between late and early bilinguals reaching high PL-L2. That means the experience of L2 acquisition may shape brain function and structure in bilinguals. We hence hypothesized that early and late bilinguals may have a difference not only in dFC among the language network regions, such as the IFG, the MFG, and the middle temporal gyrus (MTG), but also in the dynamic topological properties of the language network.

## Materials and Methods

### Subjects

**FIGURE 1:**
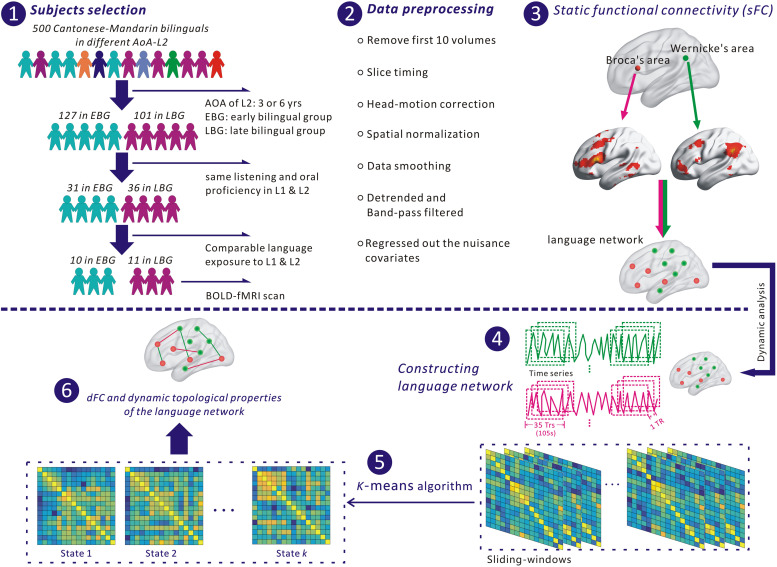
The flowchart for analyzing the language network in this study. (1) Selection of subjects. (2) Preprocessing fMRI data with SPM 12 and DPABI. (3) Identifying nodes of the language network based on Broca’s and Wernicke’s areas. (4) Extracting the time series of each node for constructing the language network. (5) Identifying dFC states of the language network with a *k*-means algorithm. (6) Estimating dynamic functional connectivity (dFC) and dynamic topological properties of the language network.

This study recruited early and late Cantonese–Mandarin bilinguals. Figure 1 shows the flowchart of this study, including the procedure of selecting subjects. Through questionnaires and interviews, we selected the subjects from a pool containing 500 volunteers of Cantonese–Mandarin bilinguals based on their age of acquisition in second language (AoA-L2), the performance of listening and oral proficiency level in L1 (PL-L1) and L2 (PL-L2), and the exposure period to L1 and L2. At the beginning, each of these 500 subjects attended an interview and finished a questionnaire to confirm the age of acquisition in L2 (AoA-L2). Afterward, we excluded 272 subjects whose AoA-L2 was more than 7 years old from further experiment and kept the 228 subjects for the next steps in the experiment. We classified these 228 bilinguals into two groups: (i) the early bilingual group (EBG) including 127 early bilinguals who acquired L2 at about 3.5 years old and (ii) the late bilingual group (LBG) including 101 late bilinguals who acquired L2 at about 6.5 years old. The classification steps are described as follows. These 228 subjects took part in the listening and oral PL assessments of L1 and L2. The assessment of listening PL includes two parts: one is a self-report with 10 linguistic questions through the European Framework of Reference (CEFR) for Languages, and the other is to estimate answer accuracy to the listened stories in L1 and L2 with five probe questions. The 10 linguistic questions and 5 probe questions to the story were randomly presented to all 228 subjects. Subjects who answered correctly no less than six linguistic questions and four questions to the story were assigned to the high listening PL. For the oral PL, three language experts were invited to grade the subject’s ability of using L1 or L2 by the standard of CEFR from A1 (breakthrough) to C2 (mastery). The grade criteria were as follows: the competence level of basic users was assessed in A1 or A2, independent users were assessed in B1 or B2, and proficient users were assessed in C1 or C2. After listening and oral PL assessments of L1 and L2, we chose 67 bilinguals who had comparable high listening and oral PL in L1 and L2. At last, a seven-point scale was used to evaluate all bilinguals’ language exposure in their different age grades (“1” represents only exposed to L1, “7” represents only exposed to L2, and “4” represents the same language exposure to L1 and L2). Based on these selection criteria, two bilingual groups, the EBG and the LBG, were obtained for the subsequent fMRI scan. We have selected 10 subjects in the EBG and 11 subjects in the LBG based on their AoA-L2 and other language measurements. The detailed information about their AoA-L2, listening and oral PL in L1 and L2, and language exposure to L1 and L2 of all 21 subjects is listed in Supplementary Table S1 (Supplementary Material).

All subjects were right-handed according to the Edinburgh Handedness Inventory (EHI) scores. No subjects reported current or history of neurological or psychiatric disorders or brain injury. Written notification of informed consent was collected from each subject before the study. The protocols were approved by the Review Board of South China Normal University.

### Image Acquisition

All MRI data were acquired on a 1.5-T Philips Achieva Nova Dual MR scanner at the Department of Radiology, Huangpu Clinical Medical Center, First Affiliated Hospital of Sun Yat-sen University, Guangzhou, China. The functional images were obtained using a single-shot gradient-echo echo-planner imaging (GE-EPI) sequence with the following parameters: repetition time (TR) = 3,000 ms, echo time (TE) = 40 ms, flip angle (FA) = 90°, field of view (FoV) = 240 mm × 240 mm, matrix size = 64 × 64, slice thickness = 4 mm without interslice gap, and 180 volumes acquired in 9 min. We also obtained high-resolution T1-weighted brain structural images of detailed anatomy for each subject using a rapid interference phase gradient echo flip recovery pulse sequence (TR/TE = 9.6 ms/3.8 ms, FA = 8°, FoV = 256 × 256 mm, data matrix = 288 × 288, slice thickness = 1 mm, voxel size = 1 mm^3^) and 176 sagittal slices (from left to right) covering the whole brain. To minimize head movements during testing, which could damage the quality of images, we used a pair of foam paddings on both sides of the head. During the rs-fMRI scanning, each subject was inquired to lie quietly in the scanner, close his or her eyes, remain wake, and not think about anything.

### Preprocessing Data

Functional imaging data were preprocessed in SPM 12^1^ and DPABI^2^. The first 10 volumes were discarded to allow for the MRI signal to approach steady state. We then preprocessed slice timing for the remaining 170 volumes to account for the acquisition time delay among slices before realigning to the first volume for head-motion correction. Subsequently, the function images were spatially normalized to the standard MNI-152 template and resampled to a voxel size of 3 mm × 3 mm × 3mm with a kernel of full-width at half-maximum (FWHM) of 8 mm. Last, the data were signal-linear-detrended and band-pass-filtered (0.01–0.08 Hz). In the calculations, we finally regressed out the nuisance covariates including the head-motion effect derived from the Friston 24 correction (Friston 24-parameter model, white matter signal, and cerebrospinal fluid signal) within each voxel in the whole brain. Due to the controversy of regressing out the global signal in rs-fMRI analysis (Fox et al., 2009; Murphy et al., 2009), we did not regress out the global signal in this study.

### Constructing the Language Network

Two regions-of-interest (ROIs), Broca’s and Wernicke’s areas, were selected in the MNI space and were used to define the language network with a standard seed-voxel approach. Particularly, we defined two ROIs with 3-mm-radius spheres as seeds based on automated anatomical labeling (AAL) atlas (Tzourio-Mazoyer et al., 2002). A previous study (Tomasi and Volkow, 2012) used these two seeds and applied a seed-voxel approach to identify the language network. One ROI was close to the left pars triangularis (MNI coordinate: *x* = −53, *y* = 20, *z* = 15) representing the Broca’s area, and the other ROI was close to the left supramarginal gyrus (MNI coordinate: *x* = −51, *y* = −51, *z* = 30) representing the Wernicke’s area. The averaged time series of these two ROIs during the whole scanning was extracted from each subject. For a given ROI, we considered it as a seed and then estimated the static functional connectivity (sFC) and Pearson’s correlation coefficient *r*, between the selected ROI and each voxel in the whole brain. Hence, we obtained the sFC maps based on Broca’s and Wernicke’s areas. Next, the Fisher’s *r*-to-*z* transform was adopted to convert these sFC correlation maps into *z*-value maps for the following statistical analysis.

One sample *t*-test was used to detect the group-level sFC among 21 subjects examined in this study. We defined the clusters with the following criteria: significant threshold *p* < 0.01 (family-wise error, FWE-corrected), and the number of voxels in each cluster exceeds 50 voxels based on a gray matter template of more than 90% probability in SPM 12. In this way, we determined the clusters that strongly connected to the Broca’s and Wernicke’s areas. For a given cluster in the sFC map, we selected the voxels that had statistical peak values from one sample *t*-test and drew spheres with radius = 3 mm. By considering these spheres as nodes and the sFC as the weighted edge, we built the language network in this study.

### Sliding-Windows Approach

We calculated the dynamic functional connectivity (dFC) of the language network for each subject by using a sliding-windows approach (Chang and Glover, 2010; Allen et al., 2012) in DynamicBC (Liao et al., 2014), a MATLAB toolbox for dynamic brain connectome analysis. We segmented the entire time series of each node into multiple subseries related to the sliding windows (the window length of 35 TRs or 105 s with step = 1 TR), from each of which the FC networks were constructed. The reason for selecting a window length of 35 TRs is because the sliding-windows correlation analysis with a short window length could induce artificial fluctuations in estimating dFC (Lindquist et al., 2014; Hindriks et al., 2016; Liegeois et al., 2017). Hence, we selected the window length of about 100 s, which was suggested by previous studies (Liu et al., 2018; Pang et al., 2018; Li et al., 2019; Chen et al., 2020). According to the above analysis, we obtained 136 subseries in the sliding windows for each subject. In each sliding window, we calculated the Pearson’s correlation coefficient *r* between the subseries of any two nodes to identify the dFC of the language network in this study.

### Identifying dFC States

We used a *k*-means clustering algorithm to cluster all sliding windows into several separate clusters (i.e., connectivity states, dFC states) based on the Euclidean distance metric. Earlier studies (Allen et al., 2012; Yang et al., 2014) suggest that brain spontaneous activity is changing over time. These changes in blood oxygenation level dependent (BOLD) signal showing several similar connectivity patterns in the brain are considered as dFC states. In the calculations, we extracted the dFC of the language network in a given sliding window and then arranged all sliding windows in sequence to constitute the dynamic connectivity matrix for each subject. Since there were *N* nodes in each sliding window and 136 sliding windows in each subject, the dynamic connectivity matrix included 136 rows (136 sliding windows) and 21 × (*N* × *N* – *N*)/2 columns (all dFC of 21 subjects in 1 sliding window). Finally, the *k*-means algorithm (*k* from 2 to 12) was applied to divide all sliding windows into several dFC states based on the dynamic connectivity matrix.

### Criteria for k-Means Solution

The silhouette score (Rousseeuw, 1987) was used to check the better solution of the *k*-means algorithm. It is applied to calculate the separation distance between any two resulting clusters. The silhouette score near 1 corresponds to the fact that the sample is distant from its neighboring clusters, while the silhouette score near 0 means the decision boundary is very close between these two neighboring clusters, and negative means that the sample might have been assigned to the wrong cluster. We selected an optimal *k* solution based on the silhouette score that in this solution was significantly higher than it was in the last *k* solution.

### dFC of the Language Network

After identifying several optimal dFC states, we studied the difference in dFC between the EBG and the LBG. Particularly, we first extracted all dFCs in 136 sliding windows for each subject. Then, we averaged those dFCs that are in the same dFC state for each subject. At last, the difference in dFC of the language network between the EBG and the LBG can be studied in each dFC state.

### Dynamic Topological Properties of the Language Network

We studied the difference in the dynamic topological properties of the language network between the EBG and the LBG. The dynamic topological properties were estimated using GRETNA^3^. We calculated the dFC and generated 136 symmetric matrices for each subject. In this study, each dFC should satisfy a threshold of significance level of *p* < 0.05 (FWE-corrected) compared to others (Cruse et al., 2011). Based on the dFC in each sliding window, we estimated the dynamic topological properties, including the clustering coefficient (*C*_w_), the characteristic path length (*L*_w_), the global efficiency (*E*_glob_), and the local efficiency (*E*_loc_) by using the graph theory for all subjects. The description of these four parameters is listed in Supplementary Table S3. Afterward, we determined the difference in the dynamic topological properties of the language network between the EBG and the LBG in each dFC state. The definitions and descriptions of the above parameters can be also found in Rubinov and Sporns (2010).

### Statistical Analysis

A nonparametric permutation *t*-test was used to determine the difference in dFC and dynamic topological properties of the language network between the EBG and the LBG. Briefly, for a given parameter (dFC, *C*_w_, *L*_w_, *E*_glob_, and *E*_loc_), we randomly paired its values between the EBG and the LBG, and then generated a new group. Subsequently, we recalculated the mean value of this new group. This permutation was repeated 5,000 times to acquire the empirical distribution of the difference in all new groups. In the calculations, we set a significant level at *p* < 0.05 to determine the significant difference between the EBG and the LBG at 95% of the empirical distribution in a two-tailed *t*-test. Given the small sample size of the subjects in our study, we also calculated the corresponding effect size (Cohen *d*) according to Cohen (2013).

## Results

### Behavioral Tests

Neither age nor gender had significant differences between the EBG and the LBG. For the language test, no significant between-group difference was found in the scores of L1 and L2 listening PLs, L1, and L2 Oral PLs, and language exposure level in separate grades after 6 years old. The detailed information about the behavioral tests between the EBG and the LBG is listed in Supplementary Table S2 (Supplementary Material).

### Language Network

**FIGURE 2:**
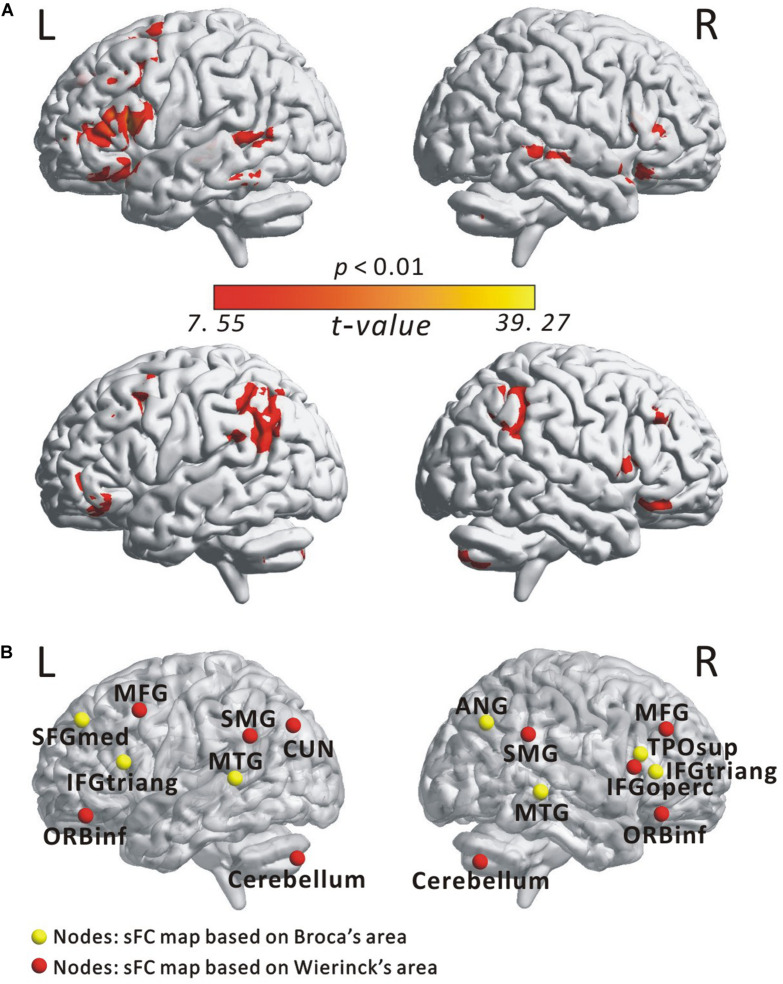
Spatial distribution of temporal correlations for Broca’s and Wernicke’s areas estimated using static functional connectivity (sFC). **(A)** sFC map of Broca’s and Wernicke’s areas. The color bar represents *t*-value (*p* < 0.01, FWE-corrected). **(B)** Nodes in the language network based on the sFC maps. Abbreviation: MTG, middle temporal gyrus; IFGtriang, inferior frontal triangular part; ORBinf, inferior frontal orbital; SFGmed, superior medial frontal gyrus; TPOsup, temporal pole (superior temporal gyrus); MFG, middle frontal gyrus; ANG, angular gyrus; IFGoperc, inferior frontal opercularis; SMG, supramarginal gyrus; CUN, cuneus; L(R), left (right) hemisphere.

Figure 2A illustrates the determined sFC maps based on Broca’s and Wernicke’s areas (*p* < 0.01, FWE-corrected and cluster size > 50 voxels) for all subjects. We detected seven clusters in which the time series was significantly positively correlated with that of Broca’s area, including the bilateral MTG, the inferior frontal triangular part (IFGtriang), the left superior medial frontal gyrus (SFGmed.L), the right angular gyrus (ANG.R), and the temporal pole (superior temporal gyrus, TPOsup.R). Meanwhile, we also detected 10 clusters in which the time series was meaningfully positively correlated with that of Wernicke’s area, including the bilateral supramarginal gyrus (SMG), the inferior frontal orbital (ORBinf), the cerebellum, the left cuneus (CUN.L), the middle frontal gyrus (MFG.L), the right middle frontal gyrus (MFG.R), and the inferior frontal opercularis (IFGoperc.R). In the calculations, we found no cluster in which the signals were significantly negatively correlated with that of either Broca’s or Wernicke’s area after applying FWE correction. More detailed information of these clusters is shown in Table 1. Finally, we detected the voxels with statistical peak values of *t*-test among these 17 clusters to draw spheres (3-mm radius) as the nodes and to construct the language network, which can be found in Figure 2B.

**TABLE 1:** Cluster locations and peak coordinates in the language network corresponding to the static functional connectivity (sFC) based on Broca’s and Wernicke’s areas.

Seed region	Cluster location	Cluster size	BA	*t*-value in peak voxel	Peak coordinate in MNI space		
					*x*	*y*	*z*
Broca’s area	IFGtriang.L	1252	48	39.27	−51	21	15
	TPOsup.R	128	38	14.61	48	21	18
	IFGtriang.R	80	14	11.53	57	30	9
	MTG.L	307	22	11.20	−60	−42	6
	MTG.R	153	21	10.43	69	−36	−3
	SFGmed.L	99	9	10.07	0	45	39
	ANG.R	51		8.93	39	−66	36
Wernicke’s area	SMG.L	786	40	37.17	−54	−51	30
	SMG.R	391	48	13.19	51	−42	30
	MFG.L	157	9	12.58	−42	12	45
	ORBinf.R	58		12.15	51	33	−15
	Cerebellum.R	170		11.48	33	−69	−42
	ORBinf.L	191	47	10.58	−39	42	−15
	IFGoperc.R	59	44	10.43	57	18	12
	MFG.R	115	46	10.31	42	36	33
	CUN.L	50		9.91	−9	−75	36
	Cerebellum.L	128		9.55	−18	−78	−39

*The statistical significance is set at p < 0.01 (FWE-corrected). The clusters are listed in the order of t-value in peak voxel. BA, Brodmann’s area.*

### dFC States of the Language Network

The silhouette scores for different dFC states in the language network are shown in Supplementary Figure S1 (Supplementary Material). All sliding windows were clustered into different dFC states by the *k*-means algorithm. We determined four dFC states (i.e., State 1, State 2, State 3, and State 4) for further analysis based on the silhouette score. That is, the silhouette score (0.53) in this solution (*k* = 4) is significantly higher than that in the last solution (*k* = 3).

**FIGURE 3:**
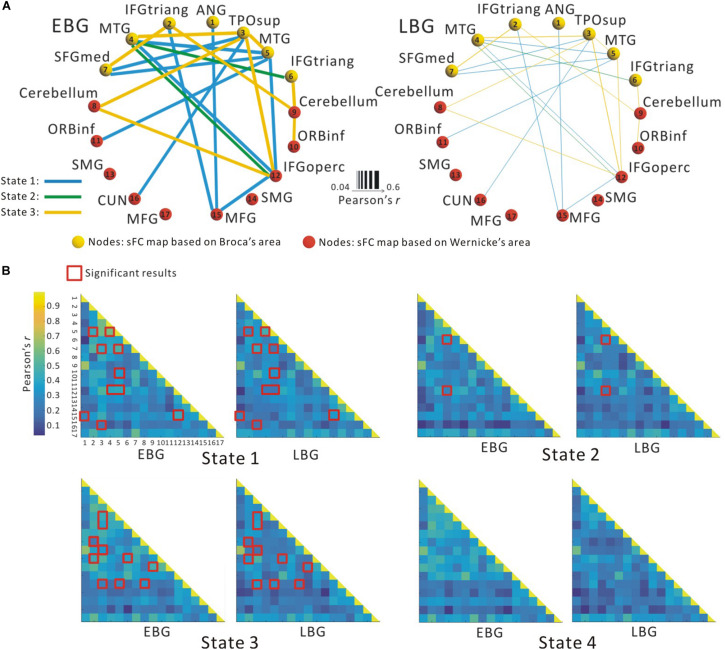
The dynamic functional connectivity (dFC) of the language network for both the early bilingual group (EBG) and the late bilingual group (LBG). **(A)** dFC of the language network in the subject group. The EBG had significantly higher dFC than the LBG (*p* < 0.05, FWE-corrected). **(B)** dFC of the language network for both the EBG and the LBG in each dFC state. The red boxes show significantly higher dFC in the EBG than the LBG (*p* < 0.05, FWE-corrected).

Figure 3 shows the between-group difference in the average dFC of the language network in each dFC state for both the EBG and the LBG. Statistical analysis (*p* < 0.05, FWE-corrected) revealed that the EBG was significantly higher in 22 dFCs compared to the LBG in three dFC states (State 1, State 2, and State 3) (Figure 3A). We found that IFGtriang.L, TPOsup.R, MTG.L, and MTG.R indicated a significantly higher dFC of the language network compared with other related regions. No significantly higher dFC was found in the LBG compared to the EBG. Detailed information about the dFC of the language network in each dFC state is listed in Table 2. The distributions of average dFC in each dFC state for both the EBG and the LBG are shown in Figure 3B.

**TABLE 2:** Dynamic functional connectivity (dFC) of the language network for both the early bilingual group (EBG) and the late bilingual group (LBG) in each dFC state.

State	dFC	Connectivity strength		*p*-value	Effect size (Cohen *d*)
		EBG	LBG		
1	ANG.R – MFG.R	0.31 ± 0.18	0.07 ± 0.15	1.2 e−3	1.45
	IFGtriang.L – MTG.R	0.45 ± 0.15	0.18 ± 0.21	2.2 e−3	1.48
	TPOsup.R − SFGmed.L	0.51 ± 0.19	0.24 ± 0.18	2.4 e−3	1.46
	TPOsup.R − CUN.L	0.36 ± 0.20	0.09 ± 0.17	1.4 e−3	1.45
	MTG.L − MTG.R	0.60 ± 0.20	0.24 ± 0.24	1.2 e−3	1.63
	MTG.L − IFGoperc.R	0.48 ± 0.28	0.02 ± 0.21	1.0 e−3	1.86
	MTG.R − SFGmed.L	0.47 ± 0.28	0.08 ± 0.27	1.6 e−3	1.42
	MTG.R − ORBinf.R	0.42 ± 0.15	0.01 ± 0.28	1.0 e−4	1.83
	MTG.R − IFGoperc.R	0.43 ± 0.19	0.14 ± 0.19	1.6 e−3	1.53
	IFGoperc.R − MFG.R	0.48 ± 0.14	0.18 ± 0.14	2.8 e−3	2.14
2	MTG.L − IFGtriang.R	0.40 ± 0.19	0.12 ± 0.18	2.4 e−3	1.51
	MTG.L − IFGoperc.R	0.37 ± 0.22	0.07 ± 0.19	1.8 e−3	1.46
3	IFGtriang.L − SFGmed.L	0.52 ± 0.17	0.27 ± 0.17	2.4 e−3	1.47
	IFGtriang.L − Cerebellum.R	0.33 ± 0.06	0.15 ± 0.17	1.8 e−3	1.41
	TPOsup.R − MTG.L	0.57 ± 0.17	0.27 ± 0.14	2.0 e−4	1.76
	TPOsup.R − MTG.R	0.55 ± 0.18	0.25 ± 0.20	1.0 e−3	1.58
	TPOsup.R − Cerebellum.L	0.44 ± 0.22	0.12 ± 0.19	8.0 e−4	1.56
	TPOsup.R − IFGoperc.R	0.50 ± 0.16	0.26 ± 0.19	2.4 e−3	1.37
	MTG.R − IFGoperc.R	0.45 ± 0.20	0.12 ± 0.23	6.0 e−4	1.53
	IFGtriang.R − Cerebellum.R	0.27 ± 0.11	0.03 ± 0.14	4.0 e−4	1.91
	Cerebellum.L − IFGoperc.R	0.41 ± 0.21	0.16 ± 0.14	1.2 e−3	1.40
	Cerebellum.R − ORBinf.R	0.32 ± 0.11	0.10 ± 0.17	8.0 e−4	1.54

*Statistical significance is set at p < 0.05 (FWE-corrected). Dynamic topological properties of the language network.*

**FIGURE 4:**
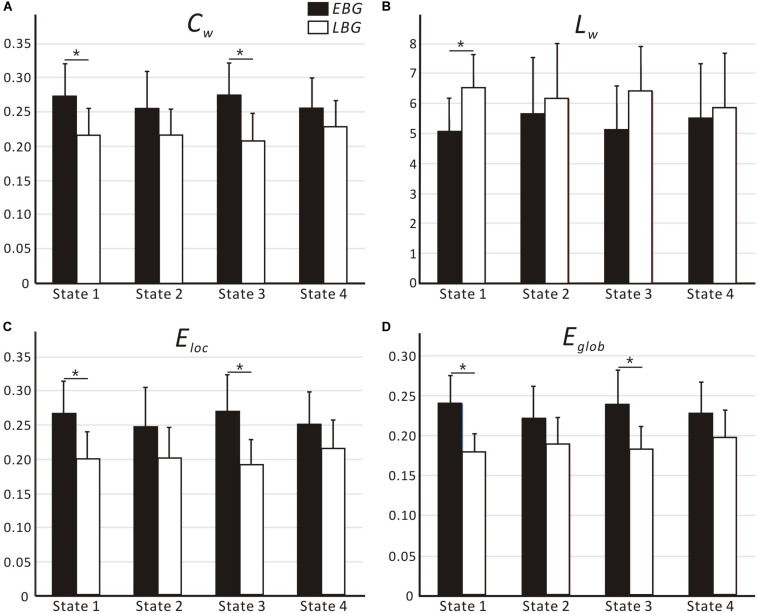
Dynamic topological properties of the language network, including **(A)** *C*_w_, **(B)** *L*_w_, **(C)** *E*_loc_ and **(D)** *E*_glob_, for both the early bilingual group (EBG) and late bilingual group (LBG) in each dFC state. For a given parameter, the bar corresponds to the mean value and the error bar to the standard deviation of a group (black: EBG, white: LBG). Abbreviations: *C*_w_, clustering coefficient; *L*_w_, characteristic path length; *E*_loc_, local efficiency; *E*_glob_, global efficiency; *p < 0.05, FWE-corrected.

Figure 4 shows the dynamic topological properties of the language network in each dFC state for both the EBG and the LBG. Statistical analysis (*p* < 0.05, FWE-corrected) revealed that compared to the LBG, the EBG had a significantly higher clustering coefficient and local and global efficiency in two dFC states (State 1 and State 3), while a significantly lower characteristic path length in one dFC state (State 1). Table 3 lists the detailed information about the statistical between-group difference and the corresponding effect size (Cohen *d*) for dynamic topological properties.

**TABLE 3:** Dynamic topological properties of the language network for both the early bilingual group (EBG) and the late bilingual group (LBG) in each of the dFC states.

Dynamic topological properties	State	EBG	LBG	*p*-value	Effect size (Cohen *d*)
Clustering coefficient (*C*_w_)	1	0.27 ± 0.05	0.22 ± 0.04	5.00 e-3*	1.10
	2	0.25 ± 0.05	0.21 ± 0.04	2.40 e-2	
	3	0.27 ± 0.05	0.21 ± 0.04	2.00 e-3*	1.33
	4	0.26 ± 0.04	0.23 ± 0.04	6.44 e-2	
Characteristic path length (*L*_w_)	1	5.10 ± 0.40	6.54 ± 1.09	6.00 e-4*	1.75
	2	5.65 ± 1.85	6.16 ± 1.43	3.18 e-1	
	3	5.12 ± 0.84	6.43 ± 1.45	1.36 e-2	
	4	5.51 ± 1.80	5.86 ± 0.79	3.20 e-1	
Global efficiency (*E*_glob_)	1	0.24 ± 0.03	0.18 ± 0.02	2.00 e-4*	2.35
	2	0.22 ± 0.04	0.19 ± 0.03	5.00 e-2	
	3	0.24 ± 0.04	0.18 ± 0.03	2.80 e-3*	1.70
	4	0.23 ± 0.04	0.20 ± 0.03	4.04 e-2	
Local efficiency (*E*_loc_)	1	0.27 ± 0.05	0.20 ± 0.04	1.20 e-3*	1.55
	2	0.25 ± 0.06	0.20 ± 0.04	2.20 e-2	
	3	0.27 ± 0.05	0.19 ± 0.04	2.20 e-3*	1.77
	4	0.25 ± 0.05	0.22 ± 0.04	4.46 e-2	

The significance is set at p < 0.05 (FWE-corrected). Each parameter is given as mean ± std. *p < 0.05 (FWE-corrected).

## Discussion

In this study, we examined the effect of AoA-L2 on the dynamic properties of the language network in Cantonese–Mandarin bilinguals. The language network for the EBG and the LBG was constructed by analyzing resting-state FC from the Broca’s and Wernicke’s areas (Figure 2, Table 1). Based on the sliding-windows method and *k*-means clustering analysis, we detected dFC and four dFC states of the language network for both early and late bilinguals. We found that the EBG showed significantly higher dFC in three states (State 1, State2, and State 3) compared to the LBG (Figure 3). In addition, the EBG had a significantly higher clustering coefficient and local and global efficiency in State 1 and State 3, but a significantly lower characteristic path length in State 1, compared to the LBG (Figure 4).

### Dynamic Language Network

We found four dFC states of the language network in both early and late bilinguals (Figure 3B result was consistent with previous studies (Zalesky et al., 2014; Betzel et al., 2016; Kabbara et al., 2017; Shine and Poldrack, 2018), which suggested that the dynamic organization of the functional network occurred at different time. The human brain is a complex and dynamic system. Dynamic organization of the functional network resulted in a well-organized way to ensure more efficient cooperation between brain regions during the brain resting state and different cognitive tasks (Raichle et al., 2001; Fox and Raichle, 2007; Sporns, 2010). A recent study (Wu et al., 2019) found more activity in subcortical areas and a connection from frontal to subcortical areas when bilinguals performed a language switching task compared to a nonverbal switching task. These results suggested a reconfigurable brain network for language and domain-general cognitive control in bilinguals. Moreover, dynamic organization of the functional network is regular, rather than random, over time, which was considered as different dFC states (Allen et al., 2014). For the language network, a set of core regions such as the IFG, the MTG, and the IPG exhibited robust responses and changed the dFC between these regions during language processing and brain resting state (Friederici and Gierhan, 2013; Fedorenko and Thompson-Schill, 2014; Liljeström et al., 2015). Chai et al. (2016) used 22 adult subjects to perform a language comprehension task and analyzed the dynamic organization of the language network. They observed that several core regions located in the left hemisphere consistently coactivated with each other and changed the dFC in language-related community over time. In this study, we found different dFC states of the language network in early and late bilinguals, which further supports the view of dynamic organization of the language network during brain resting state.

### dFC of the Language Network

Compared to the LBG, significantly higher dFC of the language network for the EBG was found in three dFC states (State 1, State 2, and State 3) (Figure 3). These results reflected that the different experience of L2 acquisition influenced the dynamic organization of the language network in bilinguals. Although late bilinguals achieve high PL-L2, early experience of L2 may still sculpture the brain representations of language in their life. Previous studies (Berken et al., 2016; Gullifer et al., 2018) explored the plastic effect of AoA-L2 on sFC between early and late bilinguals. And Berken et al. (2016) studied the AoA-L2 effect on sFC in early and late French–English bilinguals by using the rs-fMRI approach. They found higher sFC between the IFG.L and IFG.R, as well as between the IFG and those regions involved in language control in early bilinguals, compared to late bilinguals. In a previous study (Liu et al., 2017), we investigated the effect of AoA-L2 on intra- and intermodular sFC in bilinguals and revealed that the intramodular sFC in the EBG was significantly greater in semantic and phonetic modules and detected that the EBG showed significantly higher sFC between semantic and phonetic modules as well as between phonetic and syntactic modules compared to the LBG. Combining these previous findings, our results in this current study suggested that the AoA-L2 was also related to the difference in the dFC of the language network.

### dFC State of the Language Network

These four states are related to brain regional activation during language processing in bilinguals. In State 1, we found a significantly higher dFC between the superior temporal gyrus (in temporal lobe, TPOsup) and the superior medial frontal gyrus (SFGmed) in early bilinguals than that in late bilinguals. The superior temporal gyrus is related to phonetic processing (Guediche et al., 2013; Mesgarani et al., 2014). At the beginning, monolinguals start to learn L2 by listening and imitating the pronunciation of L2. Usually, late bilinguals are hard to reach the native-like pronunciation of L2 compared to early bilinguals. The AoA-L2 seems to modulate the neural activity of auditory-related regions and then induces differences in phonetic processing between early and late bilinguals. Hence, we suggested that State 1 may be related to regional neural activity for phonetic processing in early and late bilinguals. In State 2, we found a higher dFC in early bilinguals in the MTG and the IFG (IFGtriang and IFGoperc) than that in late bilinguals. Given the MTG and the IFG are mainly involved in semantic processes (Hernandez et al., 2015), we assumed that State 2 may be related to semantic processing. In State 3, we found a higher dFC in early bilinguals mainly between the cerebellum and the dominated language regions, such as IFGtriang and IFGoperc, than that in late bilinguals. The cerebellum is implicated in language control and conflict monitoring (Callan et al., 2007; Filippi et al., 2011). The increased dFC in the cerebellum in early bilinguals may indicate a more efficient language control to optimize different language processing. Accordingly, State 3 was assumed in language control. In State 4, we found no significant difference in the dFC between early and late bilinguals. By using morphosyntactic tasks, Morgan-Short et al. (2012) found no significant difference in neural activation for late L2 learners who reached high PL-L2 compared to the native speaker during syntactic processing. This result may indicate that late bilinguals with high PL-L2 also can reach native-like performance in syntactic processing. Given that no significant difference was found in State 4 on both the dFC and the dynamic topological properties, we suggested it as syntactic State 4. Our results showed that the AoA-L2 mainly influenced the dFC among the inferior frontal (IFGoperc.R and IFGtriang.R), the temporal areas (MTG, TPOsup), and the cerebellum, which suggested a difference in phonetic and semantic processing and ability of language control between early and late bilinguals.

### Dynamic Topological Properties of the Language Network

We found significant differences in the dynamic topological properties of the language network between the EBG and the LBG in different dFC states (Figure 4). Our results reflected that the basic neural mechanism of L2 acquisition in late bilinguals with high PL-L2 may differ from early bilinguals. Previous task-fMRI studies (Tham et al., 2005; Abutalebi, 2008; Sebastian et al., 2011; Consonni et al., 2013) suggested that early and late bilinguals with high PL-L2 may recruit a similar neural mechanism to process L1 and L2. Consonni et al. (2013) performed sentence comprehension and verb and noun production tasks in early and late Italian–Friulian bilinguals with high PL-L1 and -L2. They found a complete overlap of neural activations for sentence comprehension of L1 and L2 languages between early and late bilinguals. These results suggested that bilinguals with high PL-L1 and -L2 have similar neural mechanism of language processing. In this study, we constructed a common language network in early and late bilinguals at the brain resting state. However, we still found differences in the dynamic topological properties of the language network for early and late bilinguals. This may be due to the dFC states that were also related to the different stages of language processing. Bilinguals required successful communication in different language environments, while the AoA-L2 may lead to a difference in assignment of neural resources for language input, competition, inhibition, switching, and output between early and late bilinguals. Our findings provide a novel dynamic perspective to better understand the basic neural mechanism of L2 acquisition in bilinguals.

We found that the EBG had a significantly higher clustering coefficient and local and global efficiency in State 1 and State 3, but a lower characteristic path length in State 1, compared to the LBG (Figure 4). These results suggested that early bilinguals would be able to efficiently integrate some stages of language processing than late bilinguals, which may further lead to differences in neuroplasticity between early and late bilinguals. Several previous studies examined neuroplasticity in the language network after short-term L2 training (Hosoda et al., 2013; Yang et al., 2015). Yang et al. (2015) performed a novel tonal vocabulary training in 39 native English speakers. After a period of 6 weeks in L2 learning, they found that learners and nonlearners use a different language network to process tonal and lexical information of L2. In addition, successful learners showed increased activity in language-related regions such as IFG.L (BA 46), the left insula, and the right lingual gyrus, and also recruited a more cost-efficient multipath language network during L2 processing, compared to less successful learners. Similarly, Hosoda et al. (2013) performed a cohort study of L2 learning and cessation in late bilinguals. After L2 vocabulary learning, they found that late bilinguals in the training group showed increases in the gray matter volume in IFGoperc.R, as well as changed structural connectivity including the IFGoper, R-Caudate, R (CA.R), and IFGoper-SMG pathways, compared to the control group. These differences in structural plasticity were correlated with learning ability. Compared to short-term training, experience of L2 acquisition in early and late bilinguals seemed to be a kind of “long-term immersion” and also induces dynamic neuroplasticity.

### Limitations

There are several limitations in this study. First, the sample size of this study was small (only 10 subjects in the EBG and 11 subjects in the LBG), which may bias the generality of our findings. Actually, we recruited 500 subjects at the beginning. After estimating their AoA-L2, listening and oral PL, and language exposure in L1 and L2, we obtained only 21 subjects meeting the inclusion criteria. We calculated the effect size for the dFC and each of the dynamic topological properties that showed significant between-group differences. The calculation revealed that the effect sizes for dFC and dynamic topological properties were quite high (Cohen’s *d* > 1.2) in this study. In addition, recent studies (Gullifer et al., 2018; DeLuca et al., 2019; Sulpizio et al., 2020) are fast moving toward considering AoA-L2 as a continuous variable in correlational analysis with brain function and structure. In future studies, we should include more detailed behavior measurements (De Bruin, 2019) and recruit more subjects to undergo MRI scanning. Second, we identified the brain regions of the language network by estimating the conventional FC between the seed ROIs (i.e., Broca’s area and Wernicke’s area) and each voxel in the whole brain. In future studies, we should calculate the dFC between the selected ROIs and each voxel in the whole brain to identify the nodes of the language network, which may provide additional insight into the dynamic properties of language networks. Recently, Ji et al. (2016) and Zhong et al. (2019) calculated the dFC between hippocampus voxels and other voxels in the whole brain to parcellate the hippocampus and yielded more reliable hippocampus subregions. In future studies, it would be better to use the sliding-windows approach to calculate the dFC between the selected ROIs (i.e., Broca’s area and Wernicke’s area) and each voxel in the whole brain to identify the nodes of the language network. Third, this study was not a longitudinal study; hence, we cannot exclude the possibility that innate ability for language acquisition contributed to differences in the dynamic properties of the language network between the EBG and the LBG. In the future, we will consider whether different ability of language acquisition influences the dynamic properties of the language network. Fourth, we obtained rs-fMRI datasets from a 1.5-T MRI scanner, which may limit the revealing of spontaneous activity at the brain resting state. Hence, we should test our results with a high field MRI scanner. Fifth, we asked subjects to close their eyes but not think about anything during the rs-fMRI dataset collection. There were two other resting-state conditions: eyes open and fixation point. In subsequent studies, we will collect rs-fMRI datasets including both resting-state conditions. Last but not least, we used dFC and dynamic topological properties to study the dynamic properties of the language network in early and late bilinguals at the brain resting state. However, some researches (Sporns, 2010; Bola and Sabel, 2015; Braun et al., 2015; Chai et al., 2016; Giahi-Saravani et al., 2019) studied the dynamic properties of the functional network during specific tasks. In future studies, we should apply different language tasks, such as phonetic, semantic, and grammatical processing in L1 and L2, and then study the effect of the AoA-L2 on the dynamic properties of the language network during different language processing.

## Conclusion

In this study, we analyzed the dynamic properties of the language network in early and late bilinguals. We found that early bilinguals had significantly higher dynamic functional connectivity between the IFG and the MTG than late bilinguals. Compared to late bilinguals, early bilinguals displayed a higher clustering coefficient and global and local efficiency in State 1 and State 3, but a lower characteristic path length in State 2. Our result suggested the AoA-L2 is one factor affecting the dynamic properties of the language network in bilinguals. These findings may provide a dynamic perspective for understanding the neural mechanism regarding different experiences of L2 acquisition.

## Data Availability Statement

All original and processed fMRI images data related to this publication will be available upon request with a legitimate reason.

## Ethics Statement

The studies involving human participants were reviewed and approved by the Review Board of South China Normal University. The patients/participants provided their written informed consent to participate in this study.

## Author Contributions

XL, LT, XC, and RH designed the study. LT and ZL acquired the data. XL, MZ, MN, and LZ analyzed the data. XL, LT, and XC wrote the manuscript. All authors contributed to reviewing and revising the final manuscript.

## Conflict of Interest

The authors declare that the research was conducted in the absence of any commercial or financial relationships that could be construed as a potential conflict of interest.

## Footnotes

1 http://www.fil.ion.ucl.ac.uk/spm

2 http://rfmri.org/dpabi

3 http://www.nitrc.org/projects/gretna/

## References

[B1] Abutalebi J. (2008). Neural aspects of second language representation and language control. *Acta Psychol. (Amst)* 128 466–478. 10.1016/j.actpsy.2008.03.014 18479667

[B2] Allen E. A. Damaraju E. Plis S. M. Erhardt E. B. Eichele T. Calhoun V. D. (2012). Tracking whole-brain connectivity dynamics in the resting state. *Cereb. Cortex* 24 663–676. 10.1093/cercor/bhs352 23146964 PMC3920766

[B3] Allen E. A. Damaraju E. Plis S. M. Erhardt E. B. Eichele T. Calhoun V. D. (2014). Tracking whole-brain connectivity dynamics in the resting state. *Cereb. Cortex* 24 663–676. 10.1093/cercor/bhs352 23146964 PMC3920766

[B4] Archila-Suerte P. Zevin J. Hernandez A. E. (2015). The effect of age of acquisition, socioeducational status, and proficiency on the neural processing of second language speech sounds. *Brain Lang.* 141 35–49. 10.1016/j.bandl.2014.11.005 25528287 PMC5956909

[B5] Berken J. A. Chai X. Chen J. K. Gracco V. L. Klein D. (2016). Effects of early and late bilingualism on resting-state functional connectivity. *J. Neurosci.* 36 1165–1172. 10.1523/jneurosci.1960-15.2016 26818505 PMC6604829

[B6] Berken J. A. Gracco V. L. Chen J.-K. Watkins K. E. Baum S. Callahan M. (2015). Neural activation in speech production and reading aloud in native and non-native languages. *Neuroimage* 112 208–217. 10.1016/j.neuroimage.2015.03.016 25776210

[B7] Betzel R. F. Fukushima M. He Y. Zuo X.-N. Sporns O. (2016). Dynamic fluctuations coincide with periods of high and low modularity in resting-state functional brain networks. *Neuroimage* 127 287–297. 10.1016/j.neuroimage.2015.12.001 26687667 PMC4755785

[B8] Blumstein S. E. Amso D. (2013). Dynamic functional organization of language: insights from functional neuroimaging. *Perspect. Psychol. Sci.* 8 44–48. 10.1177/1745691612469021 25414726 PMC4235529

[B9] Bola M. Sabel B. A. (2015). Dynamic reorganization of brain functional networks during cognition. *Neuroimage* 114 398–413. 10.1016/j.neuroimage.2015.03.057 25828884

[B10] Braun U. Schäfer A. Walter H. Erk S. Romanczuk-Seiferth N. Haddad L. (2015). Dynamic reconfiguration of frontal brain networks during executive cognition in humans. *Proc. Natl. Acad. Sci. U.S.A.* 112 11678–11683. 10.1073/pnas.1422487112 26324898 PMC4577153

[B11] Bullmore E. T. Bassett D. S. (2011). Brain graphs: graphical models of the human brain connectome. *Annu. Rev. Clin. Psychol.* 7 113–140. 10.1146/annurev-clinpsy-040510-143934 21128784

[B12] Callan D. E. Kawato M. Parsons L. Turner R. (2007). Speech and song: the role of the cerebellum. *Cerebellum* 6 321–327. 10.1080/14734220601187733 17853077

[B13] Chai L. R. Mattar M. G. Blank I. A. Fedorenko E. Bassett D. S. (2016). Functional network dynamics of the language system. *Cereb. Cortex* 26 4148–4159. 10.1093/cercor/bhw238 27550868 PMC5066829

[B14] Chang C. Glover G. H. (2010). Time–frequency dynamics of resting-state brain connectivity measured with fMRI. *Neuroimage* 50 81–98. 10.1016/j.neuroimage.2009.12.011 20006716 PMC2827259

[B15] Chen Y. Cui Q. Xie A. Pang Y. Sheng W. Tang Q. (2020). Abnormal dynamic functional connectivity density in patients with generalized anxiety disorder. *J. Affect. Disord.* 261 49–57. 10.1016/j.jad.2019.09.084 31600587

[B16] Cohen J. (2013). *Statistical Power Analysis for the Behavioral Sciences.* Cambridge, MA: Academic Press.

[B17] Consonni M. Cafiero R. Marin D. Tettamanti M. Iadanza A. Fabbro F. (2013). Neural convergence for language comprehension and grammatical class production in highly proficient bilinguals is independent of age of acquisition. *Cortex* 49 1252–1258. 10.1016/j.cortex.2012.04.009 22622435

[B18] Cruse D. Chennu S. Chatelle C. Bekinschtein T. A. Fernández-Espejo D. Pickard J. D. (2011). Bedside detection of awareness in the vegetative state: a cohort study. *Lancet* 378, 2088–2094. 10.1016/S0140-6736(11)61224-5 22078855

[B19] De Bruin A. (2019). Not all bilinguals are the same: a call for more detailed assessments and descriptions of bilingual experiences. *Behav. Sci.* 9:33 10.3390/bs9030033 PMC6466537 30909639

[B20] Deluca V. Rothman J. Bialystok E. Pliatsikas C. (2019). Redefining bilingualism as a spectrum of experiences that differentially affects brain structure and function. *Proc. Natl. Acad. Sci. U.S.A.* 116 7565–7574. 10.1073/pnas.1811513116 30914463 PMC6462104

[B21] Fedorenko E. Thompson-Schill S. L. (2014). Reworking the language network. *Trends Cogn. Sci.* 18 120–126. 10.1016/j.tics.2013.12.006 24440115 PMC4091770

[B22] Filippi R. Richardson F. M. Dick F. Leech R. Green D. W. Thomas M. S. (2011). The right posterior paravermis and the control of language interference. *J. Neurosci.* 31 10732–10740. 10.1523/jneurosci.1783-11.2011 21775616 PMC3160463

[B23] Fox M. D. Raichle M. E. (2007). Spontaneous fluctuations in brain activity observed with functional magnetic resonance imaging. *Nat. Rev. Neurosci.* 8:700 10.1038/nrn2201 17704812

[B24] Fox M. D. Zhang D. Snyder A. Z. Raichle M. E. (2009). The global signal and observed anticorrelated resting state brain networks. *J. Neurophysiol.* 101 3270–3283. 10.1152/jn.90777.2008 19339462 PMC2694109

[B25] Friederici A. D. Gierhan S. M. (2013). The language network. *Curr. Opin. Neurobiol.* 23 250–254. 23146876 10.1016/j.conb.2012.10.002

[B26] Garbin G. Costa A. Sanjuan A. Forn C. Rodriguez-Pujadas A. Ventura N. E. (2011). Neural bases of language switching in high and early proficient bilinguals. *Brain Lang.* 119 129–135. 10.1016/j.bandl.2011.03.011 21550652

[B27] Giahi-Saravani A. Forseth K. J. Tandon N. Pitkow X. (2019). Dynamic brain interactions during picture naming. *eNeuro* 6:ENEURO.0472-18.2019. 10.1523/ENEURO.0472-18.2019 PMC6624411 31196941

[B28] Guediche S. Salvata C. Blumstein S. E. (2013). Temporal cortex reflects effects of sentence context on phonetic processing. *J. Cogn. Neurosci.* 25 706–718. 10.1162/jocn_a_00351 23281778 PMC3612392

[B29] Gullifer J. W. Chai X. J. Whitford V. Pivneva I. Baum S. Klein D. (2018). Bilingual experience and resting-state brain connectivity: impacts of L2 age of acquisition and social diversity of language use on control networks. *Neuropsychologia* 117 123–134. 10.1016/j.neuropsychologia.2018.04.037 29727624 PMC6086747

[B30] Hämäläinen S. Sairanen V. Leminen A. Lehtonen M. (2017). Bilingualism modulates the white matter structure of language-related pathways. *Neuroimage* 152 249–257. 10.1016/j.neuroimage.2017.02.081 28263923

[B31] Hernandez A. E. Hofmann J. Kotz S. A. (2007). Age of acquisition modulates neural activity for both regular and irregular syntactic functions. *Neuroimage* 36 912–923. 10.1016/j.neuroimage.2007.02.055 17490895 PMC1995424

[B32] Hernandez A. E. Woods E. A. Bradley K. A. (2015). Neural correlates of single word reading in bilingual children and adults. *Brain Lang.* 143 11–19. 10.1016/j.bandl.2015.01.010 25728012 PMC5944362

[B33] Hindriks R. Adhikari M. H. Murayama Y. Ganzetti M. Mantini D. Logothetis N. K. (2016). Can sliding-window correlations reveal dynamic functional connectivity in resting-state fMRI? *Neuroimage* 127 242–256. 10.1016/j.neuroimage.2015.11.055 26631813 PMC4758830

[B34] Hosoda C. Tanaka K. Nariai T. Honda M. Hanakawa T. (2013). Dynamic neural network reorganization associated with second language vocabulary acquisition: a multimodal imaging study. *J. Neurosci.* 33 13663–13672. 10.1523/jneurosci.0410-13.2013 23966688 PMC6618649

[B35] Ji B. Li Z. Li K. Li L. Langley J. Shen H. (2016). Dynamic thalamus parcellation from resting state fMRI data. *Hum. Brain Mapp.* 37 954–967. 10.1002/hbm.23079 26706823 PMC6867495

[B36] Kabbara A. Falou W. E. Khalil M. Wendling F. Hassan M. (2017). The dynamic functional core network of the human brain at rest. *Sci. Rep.* 7:2936. 10.1038/s41598-017-03420-6 PMC5462789 28592794

[B37] Karwowski W. Vasheghani Farahani F. Lighthall N. (2019). Application of graph theory for identifying connectivity patterns in human brain networks: a systematic review. *Front. Neurosci.* 13:585 10.3389/fnins.2019.00585 PMC6582769 31249501

[B38] Kim K. H. Relkin N. R. Lee K.-M. Hirsch J. (1997). Distinct cortical areas associated with native and second languages. *Nature* 388 171–174. 10.1038/40623 9217156

[B39] Klein D. Mok K. Chen J.-K. Watkins K. E. (2014). Age of language learning shapes brain structure: a cortical thickness study of bilingual and monolingual individuals. *Brain Lang.* 131 20–24. 10.1016/j.bandl.2013.05.014 23819901

[B40] Klein D. Zatorre R. J. Chen J.-K. Milner B. Crane J. Belin P. (2006). Bilingual brain organization: a functional magnetic resonance adaptation study. *Neuroimage* 31 366–375. 10.1016/j.neuroimage.2005.12.012 16460968

[B41] Li R. Wang L. Chen H. Guo X. Liao W. Tang Y.-L. (2019). Abnormal dynamics of functional connectivity density in children with benign epilepsy with centrotemporal spikes. *Brain Imaging Behav.* 13 985–994. 10.1007/s11682-018-9914-0 29956102

[B42] Liao W. Wu G.-R. Xu Q. Ji G.-J. Zhang Z. Zang Y.-F. (2014). DynamicBC: a MATLAB toolbox for dynamic brain connectome analysis. *Brain Connect.* 4 780–790. 10.1089/brain.2014.0253 25083734 PMC4268585

[B43] Liegeois R. Laumann T. O. Snyder A. Z. Zhou J. Yeo B. T. (2017). Interpreting temporal fluctuations in resting-state functional connectivity MRI. *Neuroimage* 163 437–455. 10.1016/j.neuroimage.2017.09.012 28916180

[B44] Liljeström M. Kujala J. Stevenson C. Salmelin R. (2015). Dynamic reconfiguration of the language network preceding onset of speech in picture naming. *Hum. Brain Mapp.* 36 1202–1216. 10.1002/hbm.22697 25413681 PMC4365727

[B45] Lindquist M. A. Xu Y. Nebel M. B. Caffo B. S. (2014). Evaluating dynamic bivariate correlations in resting-state fMRI: a comparison study and a new approach. *Neuroimage* 101 531–546. 10.1016/j.neuroimage.2014.06.052 24993894 PMC4165690

[B46] Liu J. Liao X. Xia M. He Y. (2018). Chronnectome fingerprinting: Identifying individuals and predicting higher cognitive functions using dynamic brain connectivity patterns. *Hum. Brain Mapp.* 39 902–915. 10.1002/hbm.23890 29143409 PMC6866558

[B47] Liu X. Tu L. Wang J. Jiang B. Gao W. Pan X. (2017). Onset age of L2 acquisition influences language network in early and late Cantonese-Mandarin bilinguals. *Brain Lang.* 174 16–28. 10.1016/j.bandl.2017.07.003 28711720

[B48] Mechelli A. Crinion J. T. Noppeney U. O’doherty J. Ashburner J. Frackowiak R. S. (2004). Neurolinguistics: structural plasticity in the bilingual brain. *Nature* 431 757–757. 15483594 10.1038/431757a

[B49] Mesgarani N. Cheung C. Johnson K. Chang E. F. (2014). Phonetic feature encoding in human superior temporal gyrus. *Science* 343 1006–1010. 10.1126/science.1245994 24482117 PMC4350233

[B50] Mitsuhashi T. Sugano H. Asano K. Nakajima T. Nakajima M. Okura H. (2020). Functional MRI and structural connectome analysis of language networks in japanese-english bilinguals. *Neuroscience* 431 17–24. 10.1016/j.neuroscience.2020.01.030 32027993

[B51] Morgan-Short K. Finger I. Grey S. Ullman M. T. (2012). Second language processing shows increased native-like neural responses after months of no exposure. *PLoS One* 7:e32974 10.1371/journal.pone.0032974 PMC3314650 22470434

[B52] Murphy K. Birn R. M. Handwerker D. A. Jones T. B. Bandettini P. A. (2009). The impact of global signal regression on resting state correlations: are anti-correlated networks introduced? *Neuroimage* 44 893–905. 10.1016/j.neuroimage.2008.09.036 18976716 PMC2750906

[B53] Ou J. Li W. Yang Y. Wang N. Xu M. (2020). Earlier second language acquisition is associated with greater neural pattern dissimilarity between the first and second languages. *Brain Lang.* 203:104740 10.1016/j.bandl.2019.104740 31982650

[B54] Pang Y. Chen H. Wang Y. Long Z. He Z. Zhang H. (2018). Transdiagnostic and diagnosis-specific dynamic functional connectivity anchored in the right anterior insula in major depressive disorder and bipolar depression. *Prog. Neuro Psychopharmacol. Biol. Psychiatry* 85 7–15. 10.1016/j.pnpbp.2018.03.020 29608925

[B55] Price C. J. (2010). The anatomy of language: a review of 100 fMRI studies published in 2009. *Ann. N. Y. Acad. Sci.* 1191 62–88. 10.1111/j.1749-6632.2010.05444.x 20392276

[B56] Raichle M. E. Macleod A. M. Snyder A. Z. Powers W. J. Gusnard D. A. Shulman G. L. (2001). A default mode of brain function. *Proc. Natl. Acad. Sci. U.S.A.* 98 676–682. 11209064 10.1073/pnas.98.2.676 PMC14647

[B57] Rousseeuw P. J. (1987). Silhouettes: a graphical aid to the interpretation and validation of cluster analysis. *J. Comput. Appl. Math.* 20 53–65. 10.1016/0377-0427(87)90125-7

[B58] Rubinov M. Sporns O. (2010). Complex network measures of brain connectivity: uses and interpretations. *Neuroimage* 52 1059–1069. 10.1016/j.neuroimage.2009.10.003 19819337

[B59] Sabourin L. Brien C. Burkholder M. (2014). The effect of age of L2 acquisition on the organization of the bilingual lexicon: evidence from masked priming. *Bilingualism* 17 542–555. 10.1017/s1366728913000643

[B60] Saur D. Baumgaertner A. Moehring A. Büchel C. Bonnesen M. Rose M. (2009). Word order processing in the bilingual brain. *Neuropsychologia* 47 158–168. 10.1016/j.neuropsychologia.2008.08.007 18771674

[B61] Sebastian R. Laird A. R. Kiran S. (2011). Meta-analysis of the neural representation of first language and second language. *Appl. Psychol.* 32 799–819. 10.1017/s0142716411000075

[B62] Seo R. Stocco A. Prat C. S. (2018). The bilingual language network: differential involvement of anterior cingulate, basal ganglia and prefrontal cortex in preparation, monitoring, and execution. *Neuroimage* 174 44–56. 10.1016/j.neuroimage.2018.02.010 29486320

[B63] Shine J. M. Poldrack R. A. (2018). Principles of dynamic network reconfiguration across diverse brain states. *Neuroimage* 180 396–405. 10.1016/j.neuroimage.2017.08.010 28782684

[B64] Sporns O. (2010). Networks of the brain: quantitative analysis and modeling. *Anal. Funct. Large Scale Brain Netw.* 7 9–13.

[B65] Sulpizio S. Del Maschio N. Del Mauro G. Fedeli D. Abutalebi J. (2020). Bilingualism as a gradient measure modulates functional connectivity of language and control networks. *Neuroimage* 205:116306 10.1016/j.neuroimage.2019.116306 31654763

[B66] Tham W. W. Liow S. J. R. Rajapakse J. C. Leong T. C. Ng S. E. Lim W. E. (2005). Phonological processing in Chinese–English bilingual biscriptals: an fMRI study. *Neuroimage* 28 579–587. 10.1016/j.neuroimage.2005.06.057 16126414

[B67] Tie Y. Rigolo L. Norton I. H. Huang R. Y. Wu W. Orringer D. (2014). Defining language networks from resting state fMRI for surgical planning—a feasibility study. *Hum. Brain Mapp.* 35 1018–1030. 10.1002/hbm.22231 23288627 PMC3683367

[B68] Tomasi D. Volkow N. D. (2012). Resting functional connectivity of language networks: characterization and reproducibility. *Mol. Psychiatry* 17 841–854. 10.1038/mp.2011.177 22212597 PMC3323720

[B69] Tzourio-Mazoyer N. Landeau B. Papathanassiou D. Crivello F. Etard O. Delcroix N. (2002). Automated anatomical labeling of activations in SPM using a macroscopic anatomical parcellation of the MNI MRI single-subject brain. *Neuroimage* 15 273–289. 10.1006/nimg.2001.0978 11771995

[B70] Wang J. Zuo X. He Y. (2010). Graph-based network analysis of resting-state functional MRI. *Front. Syst. Neurosci.* 4:16 10.3389/fnsys.2010.00016 PMC2893007 20589099

[B71] Wartenburger I. Heekeren H. R. Abutalebi J. Cappa S. F. Villringer A. Perani D. (2003). Early setting of grammatical processing in the bilingual brain. *Neuron* 37 159–170. 10.1016/s0896-6273(02)01150-9 12526781

[B72] Wei M. Joshi A. A. Zhang M. Mei L. Manis F. R. He Q. (2015). How age of acquisition influences brain architecture in bilinguals. *J. Neurol.* 36 35–55. 10.1016/j.jneuroling.2015.05.001 PMC5045052 27695193

[B73] Wu J. Yang J. Chen M. Li S. Zhang Z. Kang C. (2019). Brain network reconfiguration for language and domain-general cognitive control in bilinguals. *Neuroimage* 199 454–465. 10.1016/j.neuroimage.2019.06.022 31200066

[B74] Yang J. Gates K. M. Molenaar P. Li P. (2015). Neural changes underlying successful second language word learning: an fMRI study. *J. Neurol.* 33 29–49. 10.1016/j.jneuroling.2014.09.004

[B75] Yang Z. Craddock R. C. Margulies D. S. Yan C.-G. Milham M. P. (2014). Common intrinsic connectivity states among posteromedial cortex subdivisions: insights from analysis of temporal dynamics. *Neuroimage* 93 124–137. 10.1016/j.neuroimage.2014.02.014 24560717 PMC4010223

[B76] Zalesky A. Fornito A. Cocchi L. Gollo L. L. Breakspear M. (2014). Time-resolved resting-state brain networks. *Proc. Natl. Acad. Sci. U.S.A.* 111 10341–10346. 10.1073/pnas.1400181111 24982140 PMC4104861

[B77] Zhong Q. Xu H. Qin J. Zeng L.-L. Hu D. Shen H. (2019). Functional parcellation of the hippocampus from resting-state dynamic functional connectivity. *Brain Res.* 1715 165–175. 10.1016/j.brainres.2019.03.023 30910629

[B78] Zhu L. Fan Y. Zou Q. Wang J. Gao J.-H. Niu Z. (2014). Temporal reliability and lateralization of the resting-state language network. *PLoS One* 9:e85880 10.1371/journal.pone.0085880 PMC3901661 24475058

