# Supplementary material for: Dynamic Language Network in Early and Late Cantonese–Mandarin Bilinguals
Source: Front Psychol. 2020 Jun 18;11:1189. doi: 10.3389/fpsyg.2020.01189 (PMC7314931; doi:10.3389/fpsyg.2020.01189)
Supplement: Supplementary file 1 [file Table_1.DOCX]

**Supplementary materials**

**Dynamic language network in early and late Cantonese-Mandarin bilinguals**

Xiaojin Liu ^a, 1^, Liu Tu ^b, 1^, Xiaoxi Chen ^c^, Miao Zhong ^a^, Meiqi Niu ^a^, Ling Zhao ^a^, Zhi Lu ^d^ *, Ruiwang Huang ^a^ *

^a^ Center for the Study of Applied Psychology, Key Laboratory of Mental Health and Cognitive Science of Guangdong Province, School of Psychology, South China Normal University, Guangzhou 510631, P.R. China

^b^ College of Foreign Studies, Jinan University, Guangzhou 510631, P.R. China

^c^ Management School, Jinan University, Guangzhou 510631, P.R. China

^d^ Guangdong Collaborative Innovation Center for Language Research and Services, Guangdong University of Foreign Studies, Guangzhou 510420, P. R. China

^1^ Xiaojin Liu and Liu Tu contributed equally to this work.

* Correspondence should be addressed to

Ruiwang Huang, Ph.D.

School of Psychology

South China Normal University

Guangzhou 510631, P.R. China

Tel/Fax: +86 (0)20-8521 6499/ +86 (0)20-8521 6033

Email: ruiwang.huang@gmail.com

And

Zhi Lu, Ph.D.

Guangdong Collaborative Innovation Center for Language Research and Services

Guangdong University of Foreign Studies

Guangzhou 510420, P. R. China

Tel/Fax: +86 (0)20-3620 6125/ +86 (0)20- 8631 8925

Email: luzhi@gdufs.edu.cn

**Methods:**

Although Cantonese is considered as a Chinese dialect, a dramatic difference in linguistics exists between Cantonese and Mandarin. Cantonese is the dominant dialect spoken by over 80% of the population in Guangzhou, the capital of Guangdong Province, China. According to Li’s ([1990](#_ENREF_1)) statistical results, among all characters in Basic Vocabulary Table of Modern Chinese Characters ([Ye, 1987](#_ENREF_4)), only 21.5% were pronounced the same between the two. Specifically, of all the Cantonese words in a dictionary of the Guangzhou Dialect ([Rao et al., 1996](#_ENREF_3)), only 23.1% have equivalents in Mandarin. Moreover, Cantonese has its own special syntactic structures, such as post-positioned adverbials, objects and verbs. Likewise, the same rate is amazingly as low as 1.78% when the colloquial expressions of the two are compared in a handbook of translation of Cantonese and Mandarin Colloquial Expressions ([Zeng, 1982](#_ENREF_5)) . In view of the above-mentioned disparities, Mandarin and Cantonese speakers are barely able to understand each other in their verbal communications. This led us consider them as first (L1) and second (L2) language respectively in the silent, eternally expressive linguistic tasks performed in this study.

**Table S1-S2 & Figure S1**

**Table S1** Detail demographic characteristics (sex and age) for all subjects ([Liu et al., 2017](#_ENREF_2)). The listening PLs of first language (L1, Cantonese) and second language (L2, Mandarin) were assessed using the self-report (Self) method (10 questions) based on the Common European Framework of Reference (CEFR) and answering accuracy (Acc) based on the listened stories in L1 and L2 (5 probe questions). The oral PLs of L1 and L2 were assessed in two interviews, respectively, in which three language experts graded from A1 (break-through) to C2 (mastery) for each subject's usage of L1 or L2 according to a standard scale of CEFR. The language exposure, ranging from 1 (only using L1) to 7 (only using L2), in different life stages indicates the language exposure to L1 and/or L2 from birth to the time of this study. Abbreviations: Ex, language expert for L1 or L2; Self, self-report of listening PL; Acc, response accuracy; Sex, ‘0’ represents the female and ‘1’ represents the male.

| **Group** | **Subject** | **Sex** | **Age** | **Listening PLs** | | | | **Oral PLs** | | | | | | | **Language exposure across life stages** | | | | | | | |
| --- | --- | --- | --- | --- | --- | --- | --- | --- | --- | --- | --- | --- | --- | --- | --- | --- | --- | --- | --- | --- | --- | --- |
|  |  |  |  | **L1** | | **L2** | | **L1** | | | **L2** | | | |  |  |  |  |  |  |  |  |
|  |  |  |  | **Self** | **Acc** | **Self** | **Acc** | **Ex1** | **Ex2** | **Ex3** | | **Ex4** | **Ex5** | **Ex6** | **0-1 years** | **1-2 years** | **2-3 years** | **3-6 years** | **6-12 years** | **12-15 years** | **15-18 years** | **18 years-** |
| **EBG** | S1 | 0 | 22 | 7 | 5 | 8 | 5 | C2 | C2 | C2 | | C1 | C1 | C2 | 1 | 1 | 2 | 2 | 3 | 3 | 3 | 4 |
|  | S2 | 1 | 20 | 10 | 5 | 6 | 5 | C2 | C1 | C2 | | C2 | B2 | C1 | 1 | 1 | 2 | 3 | 5 | 4 | 4 | 3 |
|  | S3 | 1 | 21 | 10 | 4 | 10 | 5 | C2 | C2 | C2 | | C2 | C2 | C2 | 2 | 2 | 2 | 2 | 3 | 3 | 3 | 4 |
|  | S4 | 1 | 21 | 10 | 5 | 7 | 5 | C2 | C2 | C2 | | B2 | B2 | C1 | 1 | 1 | 1 | 2 | 3 | 3 | 2 | 4 |
|  | S5 | 0 | 20 | 6 | 5 | 7 | 5 | C1 | C1 | C2 | | C1 | C1 | C1 | 1 | 1 | 2 | 3 | 4 | 5 | 5 | 5 |
|  | S6 | 1 | 19 | 10 | 5 | 9 | 4 | C2 | C1 | C2 | | C1 | C1 | C1 | 1 | 1 | 3 | 4 | 4 | 4 | 4 | 4 |
|  | S7 | 0 | 20 | 8 | 5 | 9 | 5 | C2 | C2 | C2 | | C1 | C2 | C2 | 1 | 1 | 2 | 4 | 5 | 4 | 5 | 2 |
|  | S8 | 0 | 21 | 10 | 5 | 10 | 5 | C2 | C2 | C2 | | C1 | C1 | C2 | 1 | 2 | 3 | 3 | 3 | 4 | 4 | 6 |
|  | S9 | 1 | 27 | 6 | 3 | 6 | 5 | C2 | C2 | C2 | | B2 | C1 | C1 | 1 | 1 | 2 | 2 | 4 | 4 | 6 | 6 |
|  | S10 | 0 | 20 | 9 | 5 | 9 | 4 | C2 | C2 | C2 | | C1 | C1 | C1 | 1 | 1 | 2 | 2 | 3 | 3 | 4 | 4 |
| **LBG** | S11 | 1 | 22 | 9 | 5 | 8 | 5 | C2 | C2 | C1 | | C1 | C2 | C1 | 1 | 1 | 1 | 1 | 6 | 6 | 3 | 4 |
|  | S12 | 0 | 26 | 9 | 5 | 5 | 5 | C2 | C2 | C2 | | C1 | C1 | C2 | 1 | 1 | 1 | 1 | 4 | 4 | 5 | 5 |
|  | S13 | 0 | 20 | 8 | 5 | 7 | 5 | C2 | C2 | C2 | | C1 | C2 | C1 | 1 | 1 | 1 | 1 | 3 | 4 | 5 | 4 |
|  | S14 | 0 | 26 | 9 | 5 | 6 | 5 | C2 | C2 | C2 | | C1 | C1 | C2 | 1 | 1 | 1 | 1 | 2 | 3 | 3 | 4 |
|  | S15 | 1 | 20 | 7 | 5 | 6 | 4 | C2 | C2 | C2 | | C1 | C1 | C1 | 1 | 1 | 1 | 1 | 2 | 2 | 3 | 5 |
|  | S16 | 1 | 23 | 8 | 4 | 8 | 5 | C2 | C2 | C2 | | C2 | C2 | C1 | 1 | 1 | 1 | 1 | 4 | 4 | 4 | 5 |
|  | S17 | 1 | 24 | 9 | 4 | 9 | 5 | C2 | C2 | C2 | | C2 | C1 | C1 | 1 | 1 | 1 | 1 | 3 | 4 | 4 | 4 |
|  | S18 | 0 | 20 | 7 | 4 | 7 | 5 | C2 | C2 | C2 | | C2 | C1 | C1 | 1 | 1 | 1 | 1 | 4 | 4 | 4 | 5 |
|  | S19 | 0 | 19 | 7 | 5 | 7 | 4 | C2 | C2 | C2 | | C2 | C1 | C1 | 1 | 1 | 1 | 1 | 2 | 3 | 3 | 5 |
|  | S20 | 0 | 24 | 10 | 5 | 10 | 4 | C2 | C2 | C1 | | B2 | C1 | C1 | 1 | 1 | 1 | 1 | 3 | 4 | 4 | 5 |
|  | S21 | 0 | 22 | 8 | 4 | 7 | 5 | C2 | C2 | C2 | | C1 | B2 | C1 | 1 | 1 | 1 | 1 | 3 | 4 | 4 | 4 |

**Table S2** Demographic statistics for early bilingual group (EBG) and late bilingual group (LBG) ([Liu et al., 2017](#_ENREF_2)). All of the data are presented as mean ± SD, excepting of the ‘Oral PL’ and ‘Gender’. The values in oral PL of first language (L1, Cantonese) and second language (L2, Mandarin) were presented as a grade (Frequency), ranging from A1 (break-through) to C2 (mastery), according to six language experts.

^a^ The *p*-value was obtained using a $\chi^{2}$-test.

^b^ The *p*-value was obtained using a non-parametric permutation test.

^c^ The *p*-value was obtained using a Kendall coefficient of concordance test.

|  | **EBG**  **(*n* = 10)** | **LBG**  **(*n* = 11)** | ***p-*value** |
| --- | --- | --- | --- |
| Gender (male/female) | 5 / 5 | 4 / 7 | 0.53 ^a^ |
| Age (years old) | 21.1 ± 2.23 | 22.36 ± 2.46 | 0.10 ^b^ |
| Age of L2 acquisition (years old) | 3.30 ± 0.48 | 6.45 ± 0.52 | < 0.001^b^ |
| L1 listening PLs (Self) | 8.6 ± 1.71 | 8.27 ± 1.00 | 0.25 ^b^ |
| L1 listening PLs (Acc) | 4.7 ± 0.67 | 4.64 ± 0.50 | 0.27 ^b^ |
| L2 listening PLs (Self) | 8.1 ± 1.52 | 7.27 ± 1.42 | 0.08 ^b^ |
| L2 listening PLs (Acc) | 4.8 ± 0.42 | 4.73 ± 0.47 | 0.35 ^b^ |
| Oral PL (L1) | C2 (26)  C1 (4)  B1 (0) | C2 (28)  C1 (2)  B (0) | 0.26 ^c^, 0.10 ^b^ |
| Oral PL (L2) | C2 (8)  C1 (18)  B1 (4) | C2 (9)  C1 (22)  B1 (2) | 0.17 ^c^, 0.32 ^b^ |
| Language exposure in 2 - 3 years old | 2.10 ± 0.57 | 1.00 ± 0.00 | < 0.001^b^ |
| Language exposure in 3 - 6 years old | 2.70 ± 0.82 | 1.00 ± 0.00 | < 0.001^b^ |
| Language exposure in 6 - 12 years old | 3.70 ± 0.82 | 3.27 ± 1.19 | 0.12 ^b^ |
| Language exposure in 13 - 15 years old | 3.70 ± 0.67 | 3.82 ± 0.98 | 0.29 ^b^ |
| Language exposure in 16 - 18 years old | 4.00 ± 1.15 | 3.81 ± 0.75 | 0.25 ^b^ |
| Language exposure in 19 years old | 4.20 ± 1.23 | 4.55 ± 0.52 | 0.14 ^b^ |


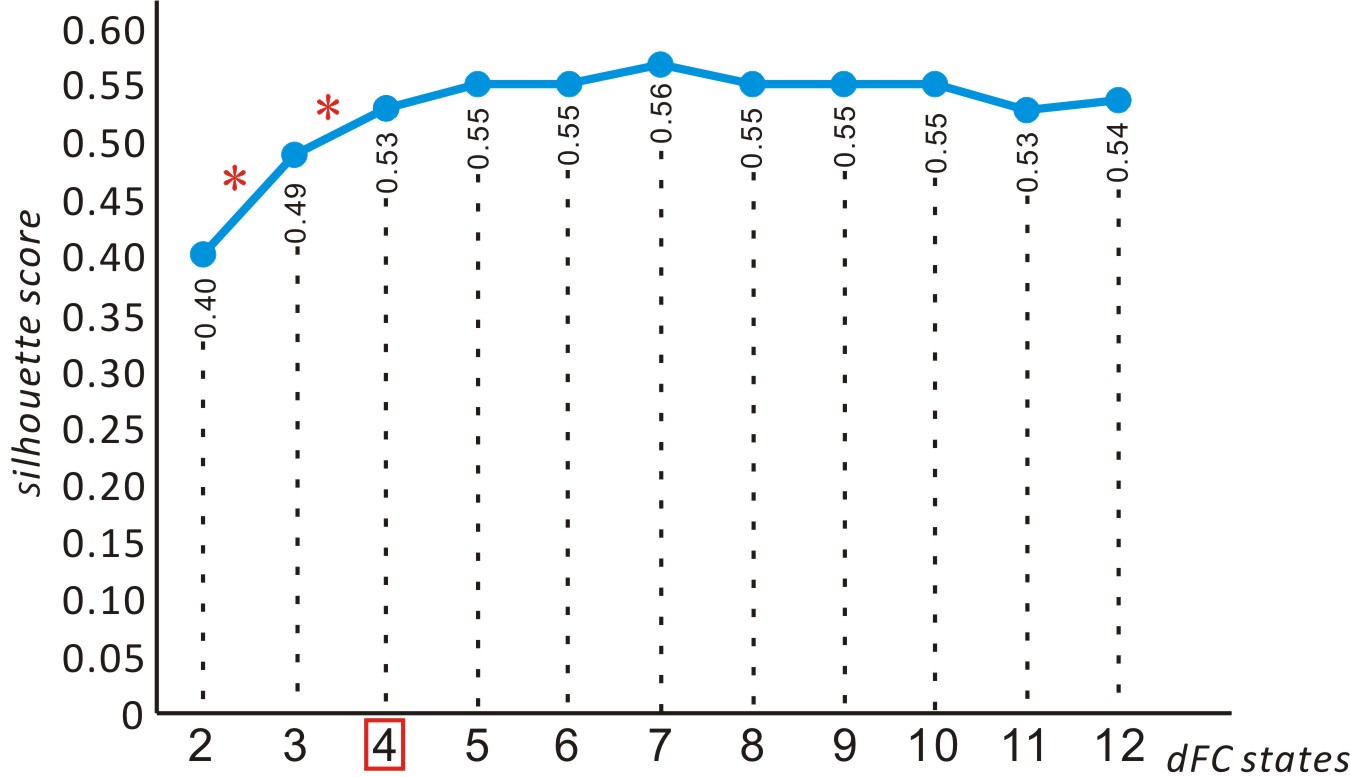


**Figure S1** The silhouette score in different dFC states. All sliding-windows in the language network for both EBG and LBG were clustered into *k* (*k* = 2, 3, … , 12) clusters, which were described as dFC states. We selected four dFC states, which showed silhouette score (0.53) in this solution significantly higher than it in last solution.

**References:**

Li, J.Z. (1990). Cantonese is independent of Chinese. *Academic Forum* 76**,** 54-76.

Liu, X., Tu, L., Wang, J., Jiang, B., Gao, W., Pan, X., Li, M., Zhong, M., Zhu, Z., and Niu, M. (2017). Onset age of L2 acquisition influences language network in early and late Cantonese-Mandarin bilinguals. *Brain and language* 174**,** 16-28.

Rao, B.C., Ouyang, J., and Zhou, W. (1996). "A Dictionary of Guangzhou Dialect". Hong Kong: Commercial Press.(In Chinese)).

Ye, C. (1987). "Basic vocabulary table of modern Chinese characters". Beijing Education Publishing House Beijing).

Zeng (1982). A handbook of translation of Cantonese and Mandarin Colloquial expressions. *Hongkong: Joint Publishing (Hong Kong) Company*.
